# Supplementary material for: Flexible simultaneous mesoscale two-photon imaging of neural activity at high speeds
Source: Nat Commun. 2021 Nov 17;12:6638. doi: 10.1038/s41467-021-26737-3 (PMC8599611; doi:10.1038/s41467-021-26737-3)
Supplement: Supplementary file 1 — Supplementary Information [file 41467_2021_26737_MOESM1_ESM.pdf]

SUPPLEMENTARY INFORMATION

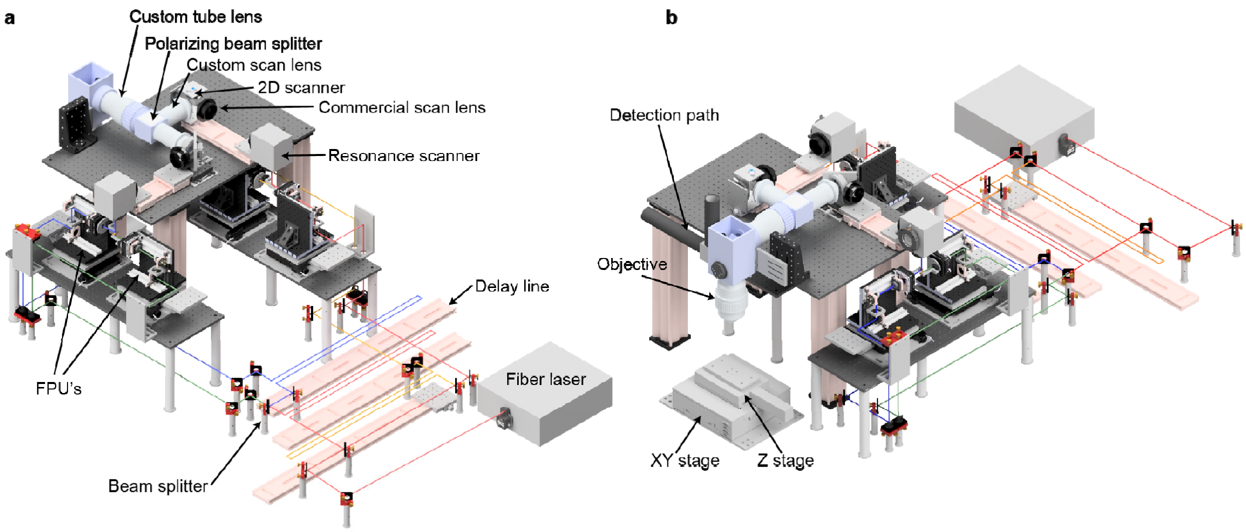

Supplementary Figure 1. Mechanical design of the Quadroscope system. a) View from the rear of the system. **b)** View from the front of the system.

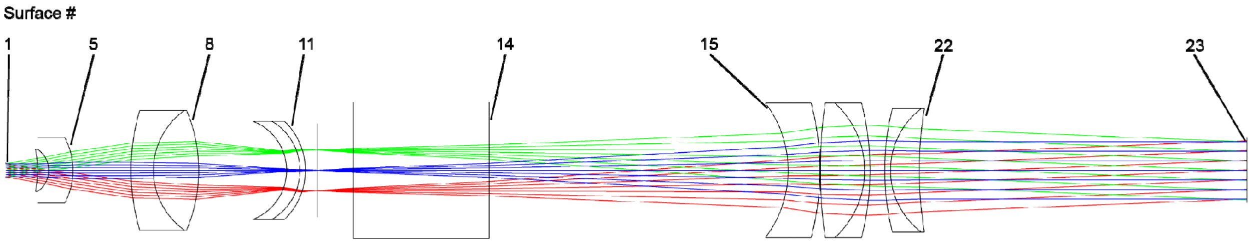

Surface #	Description	Radius (mm)	Thickness (mm)	Material	Semi-Diameter (mm)
1	2D scan mirror	Infinity	15.499		5.000
2		Infinity	1.937		10.743
3		-60.031	6.248	S-PHM53	11.600
4		-13.894	13.411	S-TIH53	11.600
5		-37.548	32.100		18.000
6		116.710	12.343	S-TIH53	33.000
7		40.204	25.041	S-LAH53	33.000
8		-83.493	48.547		33.000
9		-28.862	7.390	S-TIH53	27.000
10		-31.235	3.000	N-BK7	27.000
11		-37.279	6.600		27.000
12		Infinity	20.000		25.702
13	PBS	Infinity	75.000	N-BK7	37.500
14		Infinity	165.233		37.500
15		-60.635	17.500	N-BK7	37.000
16		-145.360	0.250		37.000
17		250.339	24.511	S-PHM52	37.000
18		-49.830	3.000	LF5	37.000
19		-146.999	8.125		37.000
20		138.454	3.175	LF5	34.000
21		42.357	15.926	S-FPL51	34.000
22		207.680	181.098		34.000
23	Objective	Infinity	0.000		19.106

Supplementary Figure 2. Lens specifications for custom scan lens and tube lens.

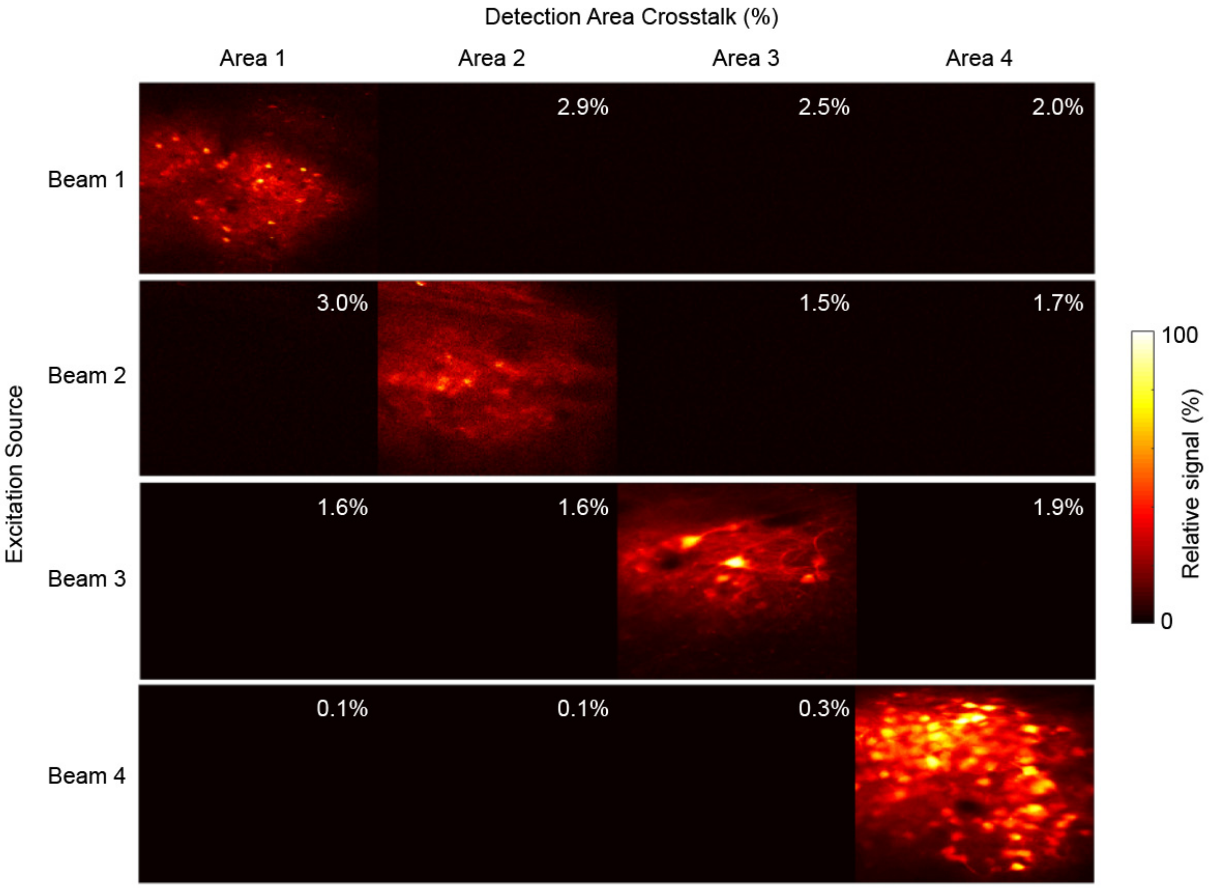

Supplementary Figure 3. Crosstalk from Spatiotemporal multiplexing/de-multiplexing. *In vivo* images of jYCaMP1s expression acquired sequentially from each excitation beam. De-multiplexed signals were assigned to each imaging area. Crosstalk for each area was calculated as the amount of detected signal relative to the main imaging area.

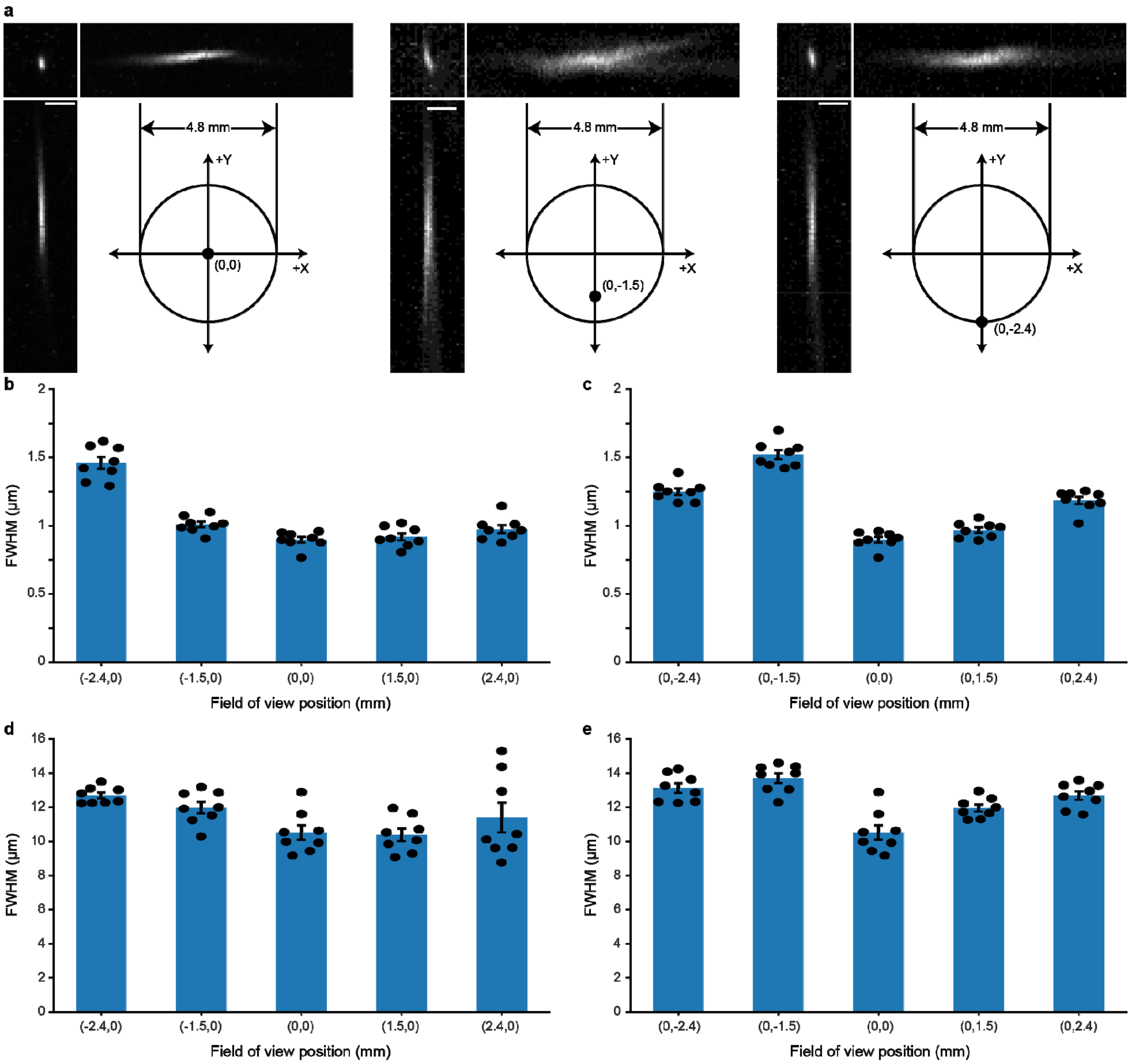

Supplementary Figure 4. Point spread function measurements across the field of view. a) Example images of 0.5 µm fluorescent beads from the center of the field of view (FOV), 1.5 mm off axis, and 2.4 mm off axis. Scale bars: 5 µm. Multiple beads have been imaged from two different sample preparations with similar results. **b,c)** Lateral full-width at half maximum (FWHM) measurements of beads along the full X axis [b] and the full Y axis [c] of the FOV. Bars represent the mean and error bars the s.e.m. from n=8 beads. **d,e)** Axial FWHM measurements of beads along the full X axis [d] and the full Y axis [e] of the FOV. The axial FWHM was measured in both the XZ and YZ planes to account for asymmetrical aberrations. Bars represent the mean and error bars the s.e.m. from n=8 beads.

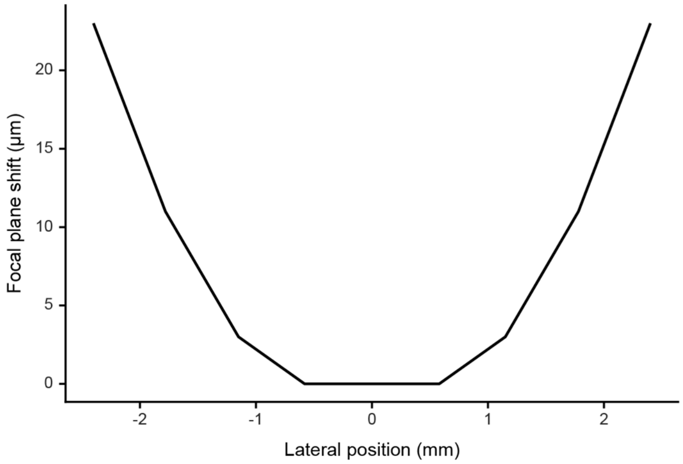

Supplementary Figure 5. Field curvature. Change in focal plane depth across the full field of view of the system as simulated in ZEMAX.

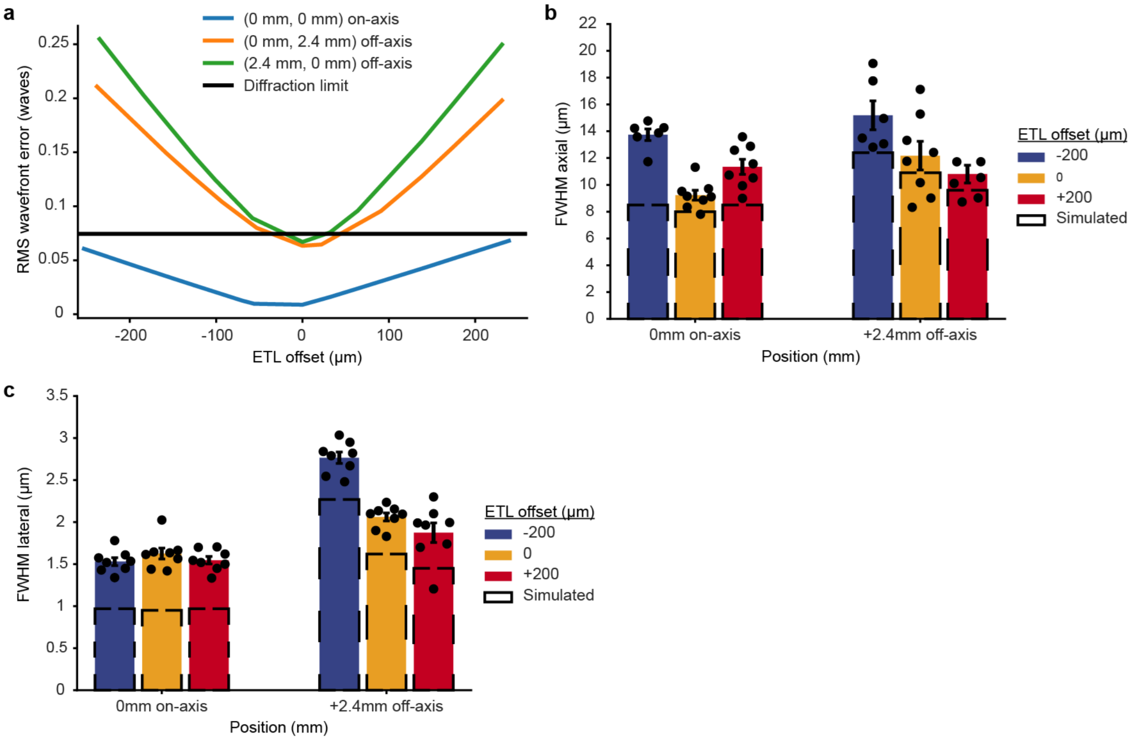

Supplementary Figure 6. Performance impact of adjusting the ETL. a) RMS wavefront error as a function of the focal plane change due to focusing of the electrically tunable lens (ETL). Performance is compared between the on axis condition and the 2.4mm off axis position in each the X and Y axis. **b and c)** Measured and simulated axial (**b**) and lateral (**c**) full width at half maximum point spread function (PSF) values. Measured values are from 0.5 µm fluorescent beads while simulated values come from the simulated Huygens PSF from ZEMAX. (**b**) The bars represent the mean and error bars the s.e.m. with n=6 beads for on axis at ETL offset -200 µm and 2.4mm off axis at ETL offset of ±200 µm and n=8 beads for all other locations. (**c**) The bars represent the mean and error bars the s.e.m. with n=8 beads for all locations.

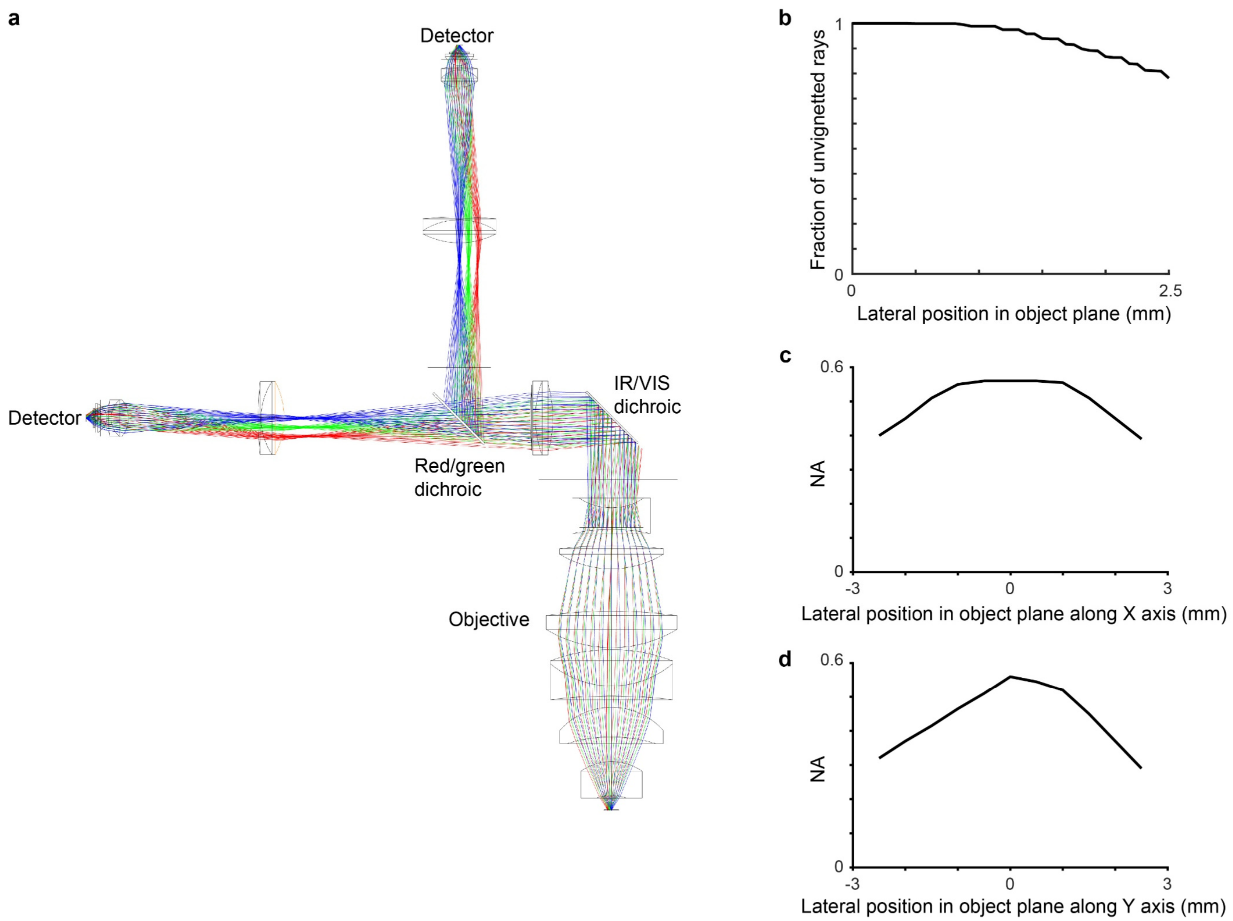

Supplementary Figure 7. Optical design and analysis of the detection system. a) Optical ray trace of the detection system. **b)** Plot of the fraction of modeled rays detected by the detector across the field of view. An efficiency of >78% was achieved across a 5 mm diameter field of view (FOV). **c and d**) Maximum numerical aperture at which all light reaches the detector as a function of position in the FOV along the x axis (**c**) and y axis (**d**).

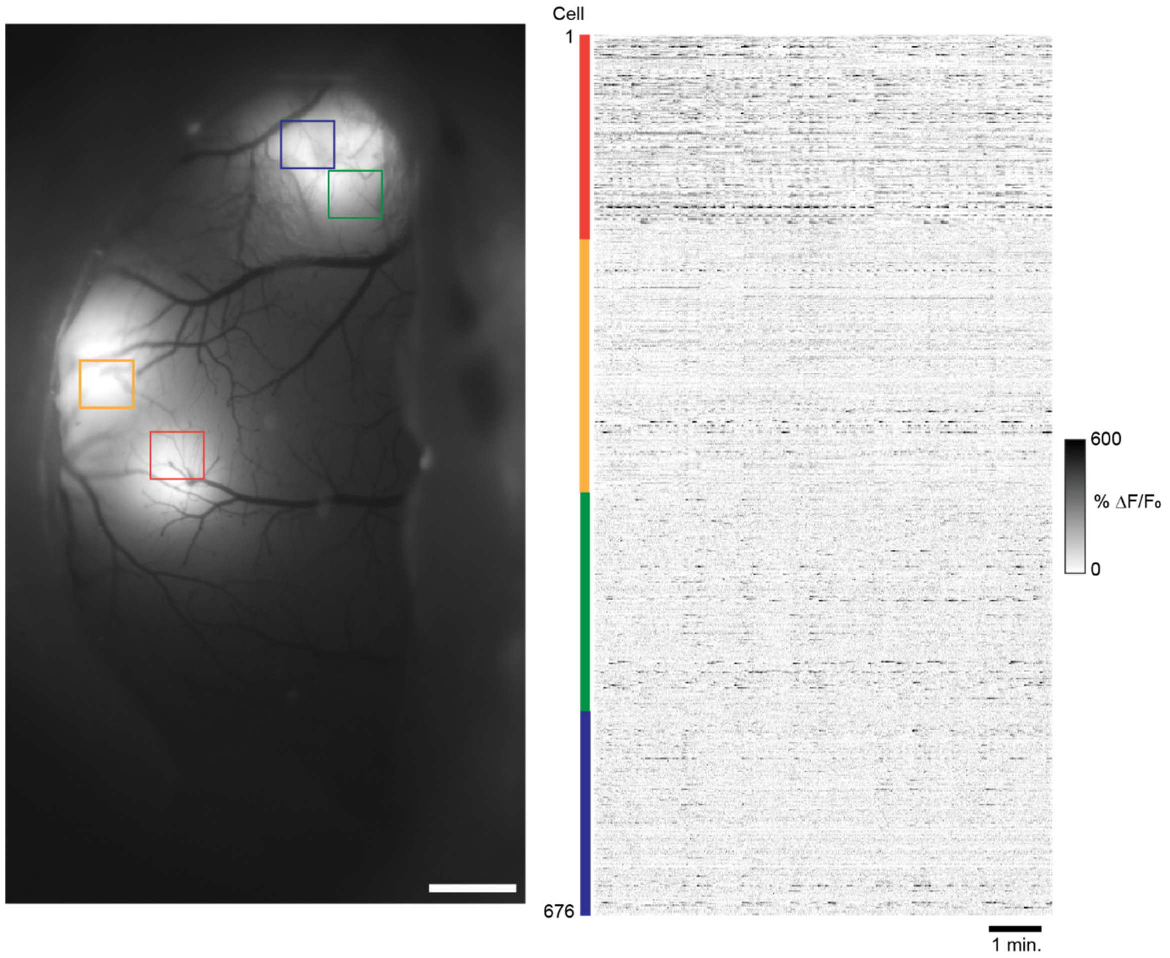

Supplementary Figure 8. Quad area two-photon calcium imaging of GCaMP7f in sensorimotor cortex in the awake mouse using a 920nm laser source. Wide-field fluorescence image of mouse sensorimotor cortex showing GCaMP7f expression through an implanted crystal skull (left). Images have been obtained from a single animal. **b)** Calcium transients in the awake animal from cortical neurons imaged across the four areas, simultaneously acquired at 30 Hz (right). Scale bars: 1 mm.

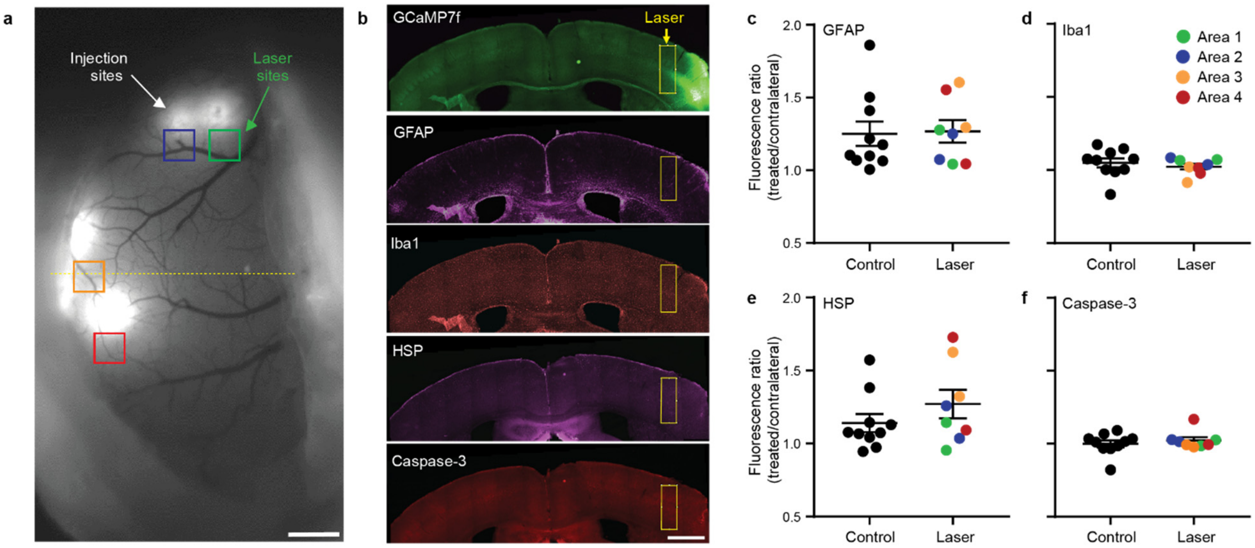

Supplementary Figure 9. Sustained Quadroscope imaging does not induce photodamage. a) Positioning of four imaging areas continuously scanned for 30 minutes at 75mW per beam .**b)** Examples of immunolabeled coronal sections on one imaging area (dotted line in [a]) with the area of laser exposure highlighted. **c-f)** No significant differences in relative fluorescence. between 8 laser-exposure areas (4 x 2 animals) and 10 control areas. Bars are mean ± s.e.m. Data points in the laser condition are color coded based on the four locations/beams. Scale bars: 1 mm.
