# Supplementary material for: Flexible simultaneous mesoscale two-photon imaging of neural activity at high speeds
Source: Nat Commun. 2021 Nov 17;12:6638. doi: 10.1038/s41467-021-26737-3 (PMC8599611; doi:10.1038/s41467-021-26737-3)
Supplement: Supplementary file 3 — Reporting Summary [file 41467_2021_26737_MOESM3_ESM.pdf]

nature research

Corresponding author(s): Jerry Chen

Last updated by author(s): Jul 22, 2021

Reporting Summary

Nature Research wishes to improve the reproducibility of the work that we publish. This form provides structure for consistency and transparency in reporting. For further information on Nature Research policies, see our Editorial Policies and the Editorial Policy Checklist.

Statistics

For all statistical analyses, confirm that the following items are present in the figure legend, table legend, main text, or Methods section.

n/a	Confirmed	
☐	☒	The exact sample size (*n*) for each experimental group/condition, given as a discrete number and unit of measurement
☐	☒	A statement on whether measurements were taken from distinct samples or whether the same sample was measured repeatedly
☐	☒	The statistical test(s) used AND whether they are one- or two-sided<br>*Only common tests should be described solely by name; describe more complex techniques in the Methods section.*
☒	☐	A description of all covariates tested
☐	☒	A description of any assumptions or corrections, such as tests of normality and adjustment for multiple comparisons
☐	☒	A full description of the statistical parameters including central tendency (e.g. means) or other basic estimates (e.g. regression coefficient) AND variation (e.g. standard deviation) or associated estimates of uncertainty (e.g. confidence intervals)
☐	☒	For null hypothesis testing, the test statistic (e.g. *F*, *t*, *r*) with confidence intervals, effect sizes, degrees of freedom and *P* value noted<br>*Give P values as exact values whenever suitable.*
☒	☐	For Bayesian analysis, information on the choice of priors and Markov chain Monte Carlo settings
☒	☐	For hierarchical and complex designs, identification of the appropriate level for tests and full reporting of outcomes
☒	☐	Estimates of effect sizes (e.g. Cohen's *d*, Pearson's *r*), indicating how they were calculated

Our web collection on statistics for biologists contains articles on many of the points above.

Software and code

Policy information about availability of computer code

Data collection: 2P data collection was performed using custom written SCOPE software which is available at http://rkscope.sourceforge.net. Zemax OpticStudio 17.5 was used to generate optical simulations of the microscope.

Data analysis: Fiji (1.53c) and MATLAB R2020a were used for bead image analysis. 2P and whisker data were processed in MATLAB R2020a. Custom analysis code can be found at https://github.com/common-chenlab/quadroscope. Our whisker tracking analysis makes use of code from https://github.com/nclack/whisk.

For manuscripts utilizing custom algorithms or software that are central to the research but not yet described in published literature, software must be made available to editors and reviewers. We strongly encourage code deposition in a community repository (e.g. GitHub). See the Nature Research guidelines for submitting code & software for further information.

Data

Policy information about availability of data

All manuscripts must include a data availability statement. This statement should provide the following information, where applicable:

- Accession codes, unique identifiers, or web links for publicly available datasets
- A list of figures that have associated raw data
- A description of any restrictions on data availability

The data that support the findings of this study are available from the corresponding authors upon reasonable request. There is a large amount of data associated with the in vivo imaging so it would be difficult to provide the full data set in a public repository.

Field-specific reporting

Please select the one below that is the best fit for your research. If you are not sure, read the appropriate sections before making your selection.

☒ Life sciences ☐ Behavioural & social sciences ☐ Ecological, evolutionary & environmental sciences

For a reference copy of the document with all sections, see nature.com/documents/nr-reporting-summary-flat.pdf

Life sciences study design

All studies must disclose on these points even when the disclosure is negative.

Sample size	Sample sizes were predetermined and reflect prior studies.
Data exclusions	Beads with an abnormal peak brightness were excluded from the point spread function analysis.
Replication	PSF measurements were performed on two separate occasions with two sample preparations each showing similar results.
Randomization	Which texture gets presented during each trial of the texture discrimination task is randomly selected from the three possibilities with a 40% probability of selecting the rewarded texture and 30% probability of selecting each of the two unrewarded textures.
Blinding	Data processing and analysis were automated making blinding of little relevance. No blinding was performed.

Reporting for specific materials, systems and methods

We require information from authors about some types of materials, experimental systems and methods used in many studies. Here, indicate whether each material, system or method listed is relevant to your study. If you are not sure if a list item applies to your research, read the appropriate section before selecting a response.

Materials & experimental systems

n/a	Involved in the study	
☐	☒	Antibodies
☒	☐	Eukaryotic cell lines
☒	☐	Palaeontology and archaeology
☐	☒	Animals and other organisms
☒	☐	Human research participants
☒	☐	Clinical data
☒	☐	Dual use research of concern

Methods

n/a	Involved in the study	
☒	☐	ChIP-seq
☒	☐	Flow cytometry
☒	☐	MRI-based neuroimaging

Antibodies

Antibodies used	Primary antibodies:<br>Mouse monoclonal anti-GFAP (Sigma-Aldrich; G3893; clone G-A-5; 1:1,000 dilution; lot:107M4792V)<br>Rabbit anti-Iba1 (Wako Chemicals; 019-19741; 1:500 dilution; lot:SKN4887),<br>Rabbit anti-cleaved caspase-3 (Asp175) (Cell Signaling; 9661; 1:250 dilution; lot:47)<br>Mouse anti-HSP70/HSP72 (Enzo Life Sciences; ADI-SPA-810-D; clone C92F3A-5; 1:400 dilution; lot:01031912).<br>Secondary antibodies:<br>Goat anti-mouse Alexa Fluor 647 (Invitrogen; A21235; 1:500 dilution; lot:2284596)<br>Goat anti-rabbit Alexa Fluor 555 (Invitrogen; A21429; 1:500 dilution; lot:1832967)
Validation	All primary antibodies show validation results on their respective manufacturer websites and were validated for use in heat damage assays in mice (Podgorski and Ranganathan 2016). Secondary antibodies Alexa Fluor 555 and 647 were validated by Invitrogen in multiple ICC/IF preparations and in several publications listed on the manufacturer website.

Animals and other organisms

Policy information about studies involving animals; ARRIVE guidelines recommended for reporting animal research

Laboratory animals	Male wild type C57BL6 mice age 12-14 weeks
Wild animals	No wild animals were used in this study.
Field-collected samples	No field collected samples were used in this study.

Ethics oversight	All procedures have been approved by the IACUC for the Charles River Campus at Boston University.

Note that full information on the approval of the study protocol must also be provided in the manuscript.
